# Expanding the Gene Expression Profiling of Drug Transporters and Drug-Metabolizing Enzymes to Include the Upper Female Reproductive Tract

**DOI:** 10.3390/pharmaceutics18050629

**Published:** 2026-05-21

**Authors:** An Le, Guru R. Valicherla, Junmei Zhang, Lin Wang, Mark K. Donnelly, Robert Bies, Lisa C. Rohan

**Affiliations:** 1 Department of Pharmaceutical Sciences, School of Pharmacy, University of Pittsburgh, Pittsburgh, PA 15213, USA; atl55@pitt.edu (A.L.); gururaghava810@gmail.com (G.R.V.); lwang@mwri.magee.edu (L.W.); 2 Magee-Womens Research Institute, Pittsburgh, PA 15213, USA; junmei.zhang@pitt.edu; 3 Department of Obstetrics, Gynecology, and Reproductive Sciences, School of Medicine, University of Pittsburgh, Pittsburgh, PA 15213, USA; 4 Division of Quantitative Methods and Modeling (DQMM), Office of Research and Standards (ORS), Office of Generic Drugs (OGD), Center for Drug Evaluation and Research (CDER), U.S. Food and Drug Administration (FDA), Silver Spring, MD 20993, USA; mark.donnelly@fda.hhs.gov; 5 Department of Pharmaceutical Sciences, School of Pharmacy and Pharmaceutical Sciences, University at Buffalo, Buffalo, NY 14214, USA; robertbi@buffalo.edu

**Keywords:** drug transporters, drug-metabolizing enzymes, gene expression, immunohistochemistry, female reproductive tract

## Abstract

**Background/Objectives**: With the ongoing efforts in supporting the discovery of novel targeted drug delivery systems for the upper region of the female reproductive tract (FRT), it is imperative to understand the local drug disposition pathways. We aim to obtain a comprehensive profile of the drug transporters and drug-metabolizing enzymes in the human ectocervix, uterus, and fallopian tubes, as these factors may substantially influence mucosal penetration, tissue exposure, drug disposition, and the risk of drug–drug interactions. **Methods:** Gene expression of 12 drug transporters and 21 drug-metabolizing enzymes was quantified using RT-qPCR. Protein expression of highly expressed transporters was assessed using immunohistochemistry (IHC). **Results**: Among the 12 transporters analyzed, the efflux transporters P-gp, BCRP, and MRP4 exhibited the highest expression across the ectocervix, endometrium, myometrium, and fallopian tubes, with P-gp consistently showing the greatest abundance in all evaluated FRT tissues. Expression of these transporters was significantly higher (6–17×) in myometrium compared with ectocervix. IHC demonstrated strong localization of P-gp, BCRP, and MRP4 to epithelial layers facing the lumen, as well as to stromal and vascular endothelial cells. For drug-metabolizing enzymes, all 21 phase I and II enzymes were detectable across the FRT, and 15 were expressed at comparatively higher levels across all tissue types. These included CYP1A1, CYP1B1, CYP2B6, CYP2C8, CYP2C19, CYP3A4, UGT1A1, UGT1A3, UGT1A4, UGT1A7, UGT1A8, UGT1A10, UGT2B4, UGT2B15, and UGT2B17. **Conclusions**: The gene expression and localization data obtained from this work may improve our understanding of drug disposition in the FRT, which will inform selection, design, and optimization of drugs intended for targeted delivery within the FRT.

## 1. Introduction

The female reproductive tract (FRT) is composed of different tissues that can be divided into two categories: upper and lower FRT [1]. These tissues support essential reproductive functions including successful pregnancy and protection against reproductive tract infections [2]. The upper FRT, including the ovaries, fallopian tubes, and uterus, is critically involved in a woman’s fertility [3]. Several reproductive health conditions, including endometritis, oophoritis, and salpingitis, affect local upper FRT, which may potentially lead to poor health outcomes and low quality of life if left untreated [4,5]. In addition, infections originating in the lower FRT local tissues (i.e., vagina) can also lead to complications in the upper FRT including tubal infertility, pre-term birth, or transmission of infections during childbirth [4,5,6]. Although substantial progress has been made in developing drug delivery systems targeting the lower FRT, there remains an increasing need for strategies that enable effective and targeted drug delivery to the upper FRT.

In order to support the ongoing efforts in the development of novel and advanced drug delivery strategies to improve upper FRT health, we aim to obtain a comprehensive profile for drug transporters and drug-metabolizing enzymes in specific compartments of this region. This information may improve our understanding of how locally delivered drugs may distribute within these distinct compartments of the upper FRT. Previous studies conducted by our group demonstrated a high prevalence of the ATP-binding cassette (ABC) transporters and phase I/II drug-metabolizing enzymes in human cervicovaginal tissue [7,8]. Among the drug transporters evaluated, P-glycoprotein (P-gp), multidrug resistance-associated protein 4 (MRP4), and breast cancer resistance protein (BCRP) have the highest expression in the human cervicovaginal tissue. Several antiretroviral agents (i.e., entry inhibitors, reverse transcriptase inhibitors, integrase inhibitors, and protease inhibitors) are substrates to these transporters, which may subsequently affect the drug exposure at the site of action [9,10]. Currently available hormonal contraceptive products are known to be inhibitors or substrates of phase I/II drug-metabolizing enzymes. Specifically, ethinyl estradiol and norethindrone are both substrates to CYP3A4 and UGT1A1 [11]. In this work, we expand the previous scope to include the upper FRT, including the uterus and fallopian tubes. Although the ovary is of interest, lack of ovary tissue availability made this analysis not feasible for the work described in this manuscript.

Besides characterizing the gene expression profile of drug transporters in the upper FRT tissues, having a better understanding of their localization in each tissue type is also imperative. Membrane transporters play a central role in determining the pharmacokinetics (PK) of many drug classes by regulating tissue entry and efflux [12]. The localization of these efflux and/or uptake drug transporters may affect the absorption, distribution, and elimination of their substrates, which subsequently impact drug disposition, safety, and efficacy. Previous studies have demonstrated the localization of efflux and uptake drug transporters in various tissues including the upper gastrointestinal tract, hepatocytes, blood–brain barrier, endothelial cells, lung, and placenta [12,13,14,15,16]. Interindividual variability in drug transporters expression and activity contributes to substantial variability in drug response. Therefore, in this study, we also aim to characterize the localization of these drug transporters in the human ectocervix, uterus, and fallopian tubes.

In this manuscript, we present a comprehensive dataset of gene expressions of 12 drug transporters and 21 drug-metabolizing enzymes in the cervical, uterine, and fallopian tube tissues as well as localization of the highly expressed drug transporters. Together with the prior work, the gene expression and localization data obtained from this work will provide a better understanding of drug distribution in the FRT. A version of this work was included in a published abstract presented at the 2025 American Society for Clinical Pharmacology and Therapeutics Annual Meeting [17].

## 2. Materials and Methods

### 2.1. Tissue Procurement and Reagents

Freshly excised human ectocervical, uterine (endometrium and myometrium), and fallopian tube tissue samples (*n* = 6 donors for each tissue type) were collected from surgical waste specimens from pre-menopausal women through the Pitt Biospecimen Core at the University of Pittsburgh under expedited approved IRB protocols (CR18110140-004, approved 2 November 2021). Due to the nature of tissue procurement, demographic information including menstrual cycle phase information, race, surgical indication, smoking status, or co-medications was not available. All collected tissue samples were evaluated by pathologists to ensure healthy anatomy. Human liver tissue samples (*n* = 2, female) were gifted by Dr. Raman Venkataramanan at the University of Pittsburgh. All the FRT tissues were de-identified and collected through an honest broker. Immediately upon collection, tissue samples were immersed in ice-cold Dulbecco’s Modified Eagle Medium (DMEM) and then transferred from the surgery site (Magee-Womens Hospital) to the laboratory (Magee-Womens Research Institute). Following collection, tissue samples were processed for experimental analyses within 2 h timeframe.

RNeasy mini kit was purchased from Qiagen (Hilden, Germany, Europe). SuperScript IV Kit was obtained from Invitrogen (Carlsbad, CA, USA). SsoFast 2× Mix was obtained from Bio-Rad (Hercules, CA, USA). Primers for qPCR were obtained from Integrated DNA Technologies (Coralville, IA, USA). Xylene, 10% buffered formalin, ethanol, paraffin embedding wax, magnesium chloride, antibody diluent, 10× antigen retrieval citra solution, and 3% hydrogen peroxide were obtained from Fisher Scientific (Hampton, NH, USA). DMEM was obtained from Corning Inc. (Corning, NY, USA). MDR1/ABCB1 (#13978), BCRP/ABCG2 (#42078), MRP4/ABCC4 (#12857) monoclonal primary antibody, 10X Tris Buffered Saline with Tween^®^ 20 (TBST), SignalStain^®^ Antibody Diluent, Signalstain^®^ Boost IHC detection reagent, and SignalStain^®^ DAB Substrate Kit were obtained from Cell Signaling Technology (Danvers, MA, USA). Cytoseal 60 was obtained from Thermo Fisher Scientific (Waltham, MA, USA). CC/mount aqueous mounting medium was obtained from Sigma-Aldrich (St. Louis, MO, USA). Hematoxylin and Eosin staining kit and hematoxylin QS were obtained from Vector Laboratories, Inc. (Burlingame, CA, USA).

### 2.2. Selection of Drug Transporters and Drug-Metabolizing Enzymes for qPCR Analysis

Selection of transporters for qPCR analysis was guided by prior reports demonstrating robust expression of ABC efflux transporters (ABCB1, ABCG2, ABCC4) in cervicovaginal epithelial tissues, in contrast to the generally low or inconsistent expression of many SLC uptake transporters in the lower FRT [7,8]. Because efflux transporters constitute the dominant barrier to luminal-to-tissue drug penetration in mucosal surfaces, we prioritized the characterization of efflux transporter expression and localization. We prioritized Phase I CYP enzymes (CYP1A1, CYP1B1, CYP2B6, CYP2C8, CYP2C9, CYP2C19, CYP2D6, CYP2E1, CYP3A4) and Phase II UGT enzymes (UGT1A1, UGT1A3, UGT1A4, UGT1A7, UGT1A8, UGT1A9, UGT1A10, UGT2B4, UGT2B7, UGT2B15, UGT2B17) because these isoforms are (i) widely recognized as the major contributors to the metabolism of drugs commonly delivered to or acting within the female reproductive tract (e.g., antiretrovirals, contraceptive steroids, antifungals), (ii) known substrates or regulators in the FDA and ICH M12 guidance for metabolism-mediated drug interactions, and (iii) previously detected at the mRNA or protein level in cervicovaginal tissues in earlier studies [7]. This targeted panel therefore focuses on DMEs with high pharmacologic relevance, well-defined substrate specificity, and potential to influence local drug exposure within the FRT.

### 2.3. Real-Time RT-qPCR

Real-time RT-qPCR was performed as previously described [18]. Ectocervical and endometrial tissue samples included mostly the epithelial layer and small portion of stromal layer. The epithelial layer of the freshly excised tissue was isolated using a Stadie-Riggs tissue slicer (Thomas Scientific, Swedesboro, NJ, USA). RNA was extracted from the cervical, uterine, and fallopian tube tissue samples following the protocol instructions from RNeasy mini kit (Qiagen, Hilden, Germany). RNA purity and concentration were evaluated using a NanoDrop spectrophotometer. The A260/A280 ratio was used to assess RNA purity, and all samples included in downstream cDNA synthesis exhibited ratios within the expected range for high-quality RNA (typically ~1.9–2.1). Samples with abnormal absorbance ratios or evidence of contamination were excluded from further analysis. Reverse transcription was carried out for the cDNA preparation using the SuperScript IV Kit. Quantitative real-time RT-PCR was conducted using the SsoFast™ 2× Mix, in the CFX Touch 96 thermocycler (Bio-Rad Laboratories, Hercules, CA, USA). The primer sequences used for real-time RT-PCR are shown in Tables S1 and S2. mRNA expression levels of different transporters and drug-metabolizing enzymes were evaluated and normalized to the levels of glyceraldehyde 3-phosphate dehydrogenase (GAPDH; housekeeping gene) using the 2^−ΔΔCq^ method in the sample.

### 2.4. Immunohistochemical (IHC) Staining

IHC staining was performed using a previously reported method [8] with minor modifications. Five-micron sections from paraffin embedded tissue were collected and then deparaffinized with xylene and hydrated in a graded ethanol series. Antigen retrieval was performed by immersing tissue sections in 1× Antigen Retrieval Citra Plus Solution (BioGenex HK086-9K, Fremont, CA, USA, pH 9.0) at 80 °C for 20 min. The slides were then incubated with 3% H_2_O_2_ for 10 min. Primary antibodies of MDR1/ABCB1 (1:1000), BCRP/ABCG2 (1:400), and MRP4/ABCC4 (1:1000) were then added onto tissue sections and incubated at 4 °C overnight. Tissue sections were then incubated with SignalStain^®^ Boost Rabbit IHC Detection Reagent (HRP, Rabbit) at room temperature for 45 min, followed by incubation with DAB (3,3′-diaminobenzidine) for detection (between 8 and 10 min). The slides were counterstained with diluted aqueous hematoxylin (approximately 20 s), followed by dehydration through a graded ethanol series and xylene. CC/mount was then applied onto the stained tissue sections. For positive controls, normal human kidney tissue was used for MDR1, normal human small intestine tissue was used for BCRP, and normal human prostate tissue was used for MRP4. For negative control, the slides were stained with secondary antibody only. Images under 20× magnification were acquired using the AxioCam software, version 4.9.1 paired with the microscope, Axioskop 40.

### 2.5. Statistical Analysis

Statistical analysis of mRNA expression between tissue types was performed with an exact Kruskal–Wallis test, followed by pairwise multiple comparisons with Dunn’s method using GraphPad Prism software version 10.4.1. A *p*-value < 0.05 was considered statistically significant. Non-parametric testing was selected due to the small sample size per group and non-normal distribution of ΔΔCt-derived expression values. All statistical comparisons were performed among FRT tissues only and were not conducted relative to liver.

## 3. Results

### 3.1. Expression of Efflux and Uptake Transporters in Cervix, Uterus, and Fallopian Tubes

#### 3.1.1. ATP-Binding Cassette (ABC) Efflux Transporters

**Figure 1:**
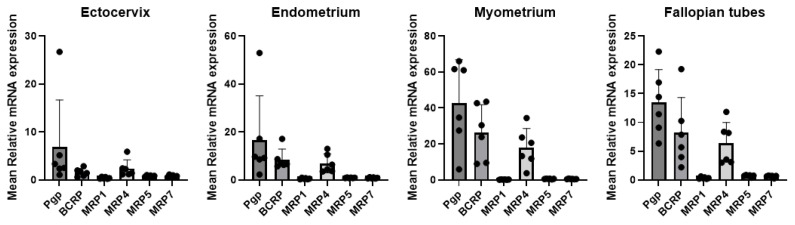
Efflux transporter expression analysis in ectocervical, endometrial, myometrial, and fallopian tube tissues (*n* = 6 donors per tissue, 3 technical replicates each). Data are shown as the mean ± standard deviation from experiments involving 3 biological replicates from each donor.

**Figure 2:**
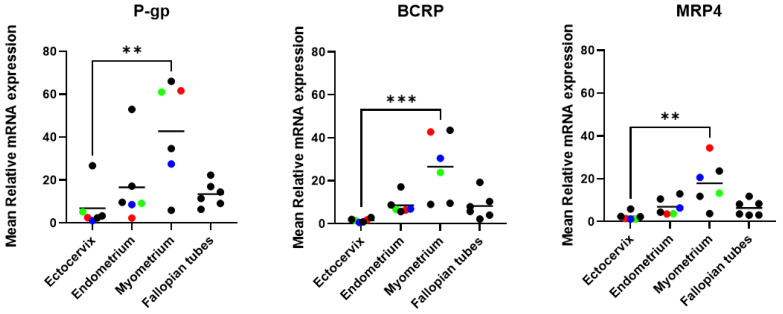
Dot plot analysis of transporter expression data in different donors (*n* = 6 donors per tissue). Data are shown as the mean ± standard deviation from experiments involving 3 technical replicates from each donor. Each black data point represents a separate donor for a specific tissue type. The data points with bright colors (red, blue, and green) indicate matched tissue samples from the same donor, with each color representing one individual donor that provided ectocervix, endometrium, and myometrium. For statistical analysis, ** indicates *p*-value < 0.01 and *** indicates *p*-value < 0.001.

mRNA expression of six ABC efflux transporters was measured in the cervix, uterus, and fallopian tubes (six donors for each tissue type). As liver tissues served only as a ΔΔCt calibrator, our interpretation focuses on relative expression patterns within the FRT. GAPDH Ct values were stable across the FRT and liver tissues (Table S3A). No statistical comparisons were performed between FRT tissues and liver; the interpretation is limited to relative expression patterns among FRT tissues. P-gp, BCRP and MRP4 transporters were found to be highly expressed in all three FRT tissue types (Figure 1). P-gp transporter showed the strongest expression among all measured efflux transporters, followed by BCRP and MRP4 as shown in Table 1 and the dot plot analysis (Figure 2). Further statistical analysis was conducted between tissue types for each efflux transporter. The expression level of P-gp, BCRP, and MRP4 was significantly higher in myometrium compared to ectocervix (*p* < 0.05), whereas no statistically significant differences were detected among the other tissue comparisons. (Figure 2). The expression level of MRP1, MRP5, and MRP7 was low in the ectocervix, endometrium, myometrium, and fallopian tubes. To further illustrate relative expression patterns within the FRT, we also provide an exploratory supplementary table presenting fold change relative to mean FRT expression for each drug transporter (Table S4).

#### 3.1.2. Solute Carrier (SLC) Uptake Transporters

The gene expression of six SLC uptake transporters varied across all three tissue types. The transporter expression levels in all three tissue types are summarized in Supplemental Data (Table S5 and Figure S1). The gene expression of these transporters was lower and more variable, with the exception of ENT2 transporters in cervical tissue. In addition, there was no clear trend of expression consistent for these transporters across all three tissue types. To further illustrate relative expression patterns within the FRT, we also provide an exploratory supplementary table presenting fold change relative to mean FRT expression for each drug transporter (Table S6).

### 3.2. Efflux Transporter Localization in Female Reproductive Tract Tissues

**Figure 3:**
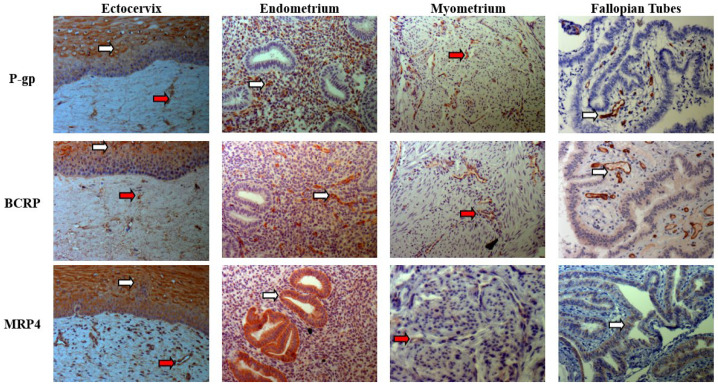
Representative images showing the localization of P-gp, BCRP, and MRP4 proteins in human ectocervical (*n =* 3), endometrial (*n =* 3), myometrial (*n =* 3), and fallopian tube tissues (*n =* 3). White arrows: epithelial cells or mucosal folds (for fallopian tubes); red arrows: vascular endothelial cells. Magnification: 20×–40×.

IHC staining was utilized to localize the proteins of highly expressed efflux transporters, P-gp, BCRP, and MRP4 in the FRT tissues. In endometrium and ectocervix, the epithelial cells facing the uterine and cervical lumen were stained positive for P-gp, BCRP, and MRP4 transporters (Figure 3). In ectocervix, the stroma was stained diffusively and qualitatively less intense than the epithelium for all the three transporters. In the endometrium, the simple columnar epithelium that lines the endometrial glands was stained qualitatively more intensely than the surrounding connective tissue. Diffuse staining was observed for the three transporters in myometrium with intense staining on the capillaries. In fallopian tubes, the capillaries in the mucosal folds were intensely stained for P-gp and BCRP, and the mucosal epithelium was diffusely stained for BCRP and MRP4. The IHC staining of positive and negative controls is shown in Figure S2.

### 3.3. Drug-Metabolizing Enzymes Expression Data

A total of six different donors for each tissue type were used to assess gene expression of 21 (10 Phase I and 11 Phase II) drug-metabolizing enzymes. The RT-PCR results for the drug-metabolizing enzymes in human ectocervix, uterus, and fallopian tube tissues are summarized in Table 2 and Figure S3. Similarly, as liver tissues served only as a ΔΔCt calibrator, our interpretation focuses on relative expression patterns within the FRT. GAPDH Ct values were stable across the FRT and liver tissues (Table S3B). For the 10 phase I drug-metabolizing enzymes evaluated, CYP1A1, CYP1B1, CYP2C8, and CYP2C19 mRNA levels showed the strongest and most consistent expression across all three FRT tissues. Among these, CYP1B1 had significantly higher expression in human ectocervix and human fallopian tubes compared to the other drug-metabolizing enzymes. We also observed moderate to high expression of CYP3A4 and CYP2B6 mRNA levels across all donors. For the 11 phase II drug-metabolizing enzymes evaluated, UGT1A1, UGT1A3, UGT1A4, UGT1A7, UGT1A8, UGT1A10, UGT2B4, UGT2B15, and UGT2B17 mRNA levels also demonstrated robust expression across all three FRT tissues. We also included UGT1A9 isoform in the gene expression study; however, the mRNA levels were not detected in any of the three FRT tissues under our experimental conditions. To further illustrate relative expression patterns within the FRT, we also provide an exploratory supplementary table presenting fold change relative to the mean FRT expression for each drug-metabolizing enzyme (Table S7).

## 4. Discussion

Drug disposition in the FRT reflects the involvement of different factors including but not limited to solubility, ionization state, epithelial permeability, transporter-mediated influx and efflux, local metabolic activity, stromal distribution, and vascular clearance. Intravaginal and intrauterine administered drugs must cross the epithelial barriers to reach the stromal layer. The presence and localization of efflux and uptake transporters at luminal surfaces may regulate mucosal drug penetration, whereas stromal and endothelial expression may suggest their involvement in redistribution toward the vasculature, thereby systemic exposure. In parallel, locally expressed drug-metabolizing enzymes may transform the molecular structure of the drug compounds and alter their stability. The primary goal of this study was to characterize the gene expression and localization of drug transporters and drug-metabolizing enzymes within the upper FRT, a region with only limited existing data. Our interpretation emphasizes cross-tissue and cross-donor patterns within the FRT and the anatomical localization of key efflux transporters, rather than absolute differences from hepatic levels. Importantly, liver gene expression values in our workflow served primarily as an internal reference anchor to control plate-to-plate variation, not as estimates of population hepatic abundance. It should also be noted that these data reflect gene expression and localization, but do not directly assess transporter activity or metabolic function. Therefore, our conclusions focus on potential implications for drug disposition rather than definitive functional effects.

Across the 12 drug transporters tested, P-gp, BCRP, and MRP4 efflux transporters were found to have high expression levels within the ectocervix, endometrium, myometrium, and fallopian tubes, with the highest relative expression observed in the myometrium. Among those three highly expressed transporters, P-gp was observed to have the highest relative expression levels and MRP4 having the lowest expression levels in ectocervix, endometrium, myometrium, and fallopian tubes. For the six uptake transporters evaluated, all have similar levels of gene expression among all three tissue types. Our findings in this study with respect to the gene expression of P-gp, BCRP, and MRP4 in human ectocervix agreed with previously reported results [19]. Zhou et al. using qPCR reported a high expression of P-gp, BCRP, and MRP4 in the human ectocervix [8]. Nicol et al. found high gene expression of P-gp and MRP4 in the human ectocervix using qPCR and reported high localization of these two transporters in the epithelium and/or submucosa [20]. Langmann et al. reported that the uterus has the highest expression of BCRP transporter in comparison to other organs of the human body [21].

Localization of the efflux transporters in the upper FRT is one of the important determinants for drug distribution. Specifically, the prominent expression of efflux transporters of P-gp, BCRP, and MRP4 in the epithelial layer of ectocervix and endometrium, as well as in the mucosal folds of the fallopian tubes, suggests they may influence luminal-to-tissue drug movement within this region. This localization is especially relevant for drugs administered via intravaginal and intrauterine routes, where these transporters could limit mucosal absorption or promote luminal clearance. In contrast, the efflux transporters localized in the stromal region of myometrium and ectocervix may be relevant to regulate drug movement between the FRT tissues and systemic circulation, either by restricting tissue uptake or facilitating efflux into the bloodstream, depending on substrate affinity. The observed expression of MRP4 transporter in the glandular epithelial cells of the endometrium is in accordance with what was observed by Ilaria Gori et al. in normal and eutopic endometrial tissue [22]. From a drug delivery perspective, localization of P-gp/BCRP/MRP4 in the ectocervical and endometrial epithelium and intense vascular/endothelial staining in myometrium and fallopian tube may suggest that substrates of these transporters may experience reduced mucosal entry and/or enhanced clearance into the lumen or vasculature. While these interpretations are derived from expression and localization data rather than functional transport studies, they may offer a useful conceptual framework. Such insights may guide candidate selection, inform formulation strategies (e.g., permeability enhancers not interacting with efflux), and support drug–drug interaction risk assessment for FRT targeted therapies. However, functional validation using known substrates and inhibitors will be essential to confirm the directionality and the extent of these transporters in modulating drug disposition within the FRT.

We identified moderate to high gene expression of 15 Phase I and II drug-metabolizing enzymes in the ectocervix, uterus, and fallopian tubes. For the 10 Phase I drug-metabolizing enzymes evaluated, CYP1A1, CYP1B1, CYP2C8, and CYP2C19 mRNA levels were found to be the highest and most consistent across all three FRT tissues. Our findings of CYP1A1 and CYP1B1 mRNA levels in the human ectocervix agree with previous work conducted by our group [7]. Given the high expression level of CYP1B1 across all donors, it is relevant to consider the role of CYP1B1 in its estrogen metabolism pathway, which produces potential carcinogenic metabolites [23,24,25,26]. In addition, CYP1B1 has also been thought to play a major role in altering tissue response to hormones and clinical response to chemotherapy [23,24,25,26]. However, our data characterized gene expression only and do not directly assess enzymatic activity; therefore, functional and clinical implications cannot be inferred without further investigation. In this study, we detected moderate to high expression of CYP3A4, CYP2B6, CYP2C8, and CYP2C19 consistently in all ectocervix donors while the previous study found minimal gene expression of these four Phase I drug-metabolizing enzymes. In addition to donor-to-donor variations, the use of different PCR methods and/or primers may have contributed to the observed differences in gene expression of these four phase I drug-metabolizing enzymes. For example, Zhou et al. used conventional PCR while this study used qPCR to quantify the expression levels of these genes [7]. In addition, the presence of CYP1A1 and CYP3A4 within the FRT provides molecular foundation for future investigation of potential drug–drug interactions involving intravaginal and intrauterine drug delivery. For instance, it was found in a phase 1 clinical study that the concomitant use of dapivirine vaginal ring and an antifungal miconazole vaginal capsule led to an increase in systemic exposure of dapivirine [27]. The observed higher systemic concentrations of dapivirine were attributed to the enzymatic inhibitory effects of miconazole on CYP1A1 and CYP3A4 [28]. For the 11 Phase II drug-metabolizing enzymes evaluated, UGT1A1, UGT1A3, UGT1A4, UGT1A7, UGT1A8, UGT1A10, UGT2B4, UGT2B15, and UGT2B17 mRNA levels were found to be consistently high across all tissue types. In the human ectocervix, UGT1A1, UGT1A7, UGT1A8, UGT1A10, UGT2B4, UGT2B15, and UGT2B17 had gene expression levels comparable to the previous study while UGT1A3 and UGT1A4 had gene expression levels higher than what was observed in the previous study [7]. As discussed above, the observed differences in gene expression between the two studies can be attributed to the use of different tissue donors, PCR methods, and/or primer sequences. It should be noted that among these drug-metabolizing enzymes, UGT2B17 mRNA levels have the highest variation in all tissue types, which agrees with previous findings [29].

Some drugs are known to interact with both drug-metabolizing enzymes and transporters, potentially causing multiple drug–drug interactions. For example, cyclosporin A (CsA) significantly inhibited hepatic and intestinal P-gp activity and increased intestinal CYP3A4 activity in renal transplant patients (while tacrolimus (FK506) or sirolimus (Rapa) did not have noticeable impact on CY3A4 or P-gp activity) [30]. Therefore, it is important in drug development to characterize gene expressions and localizations of important drug transporters and drug-metabolizing enzymes in the target tissues and evaluate their impact on metabolism and transport of drugs of interest. The gene expression profiles of drug transporters and drug-metabolizing enzymes in the upper FRT obtained in our study may improve the understanding of drug biodistribution within the ectocervix, uterus, and fallopian tubes. Specifically, these drug transporters and drug-metabolizing enzymes are known to be involved in several drug classes that are potential candidates for intravaginal delivery [31,32]. In addition, the current FDA and ICH M12 guidance recommend determining whether an investigational drug is a substrate to several predominant drug transporters and metabolizing enzymes including P-gp, BCRP, and several CYP enzymes [33,34]. In this work, we generated comprehensive gene expression data for a panel of drug transporters and drug-metabolizing enzymes, which is of critical importance for the future development of intravaginally delivered drugs. The immunohistochemical evaluations of these drug transporters demonstrate an abundance of efflux transporters in the epithelial layer and mucosal folds. While these data do not directly measure functional transport or metabolic activity, the observed localization of efflux transporters within epithelial layers and mucosal folds suggests the potential relevance for local drug disposition. Specifically, the transporters with high protein expression in the columnar and squamous epithelial cells of the ectocervix or mucosal folds of the fallopian tubes may limit mucosal penetration of a drug candidate [10]. This suggests that future studies will be required to determine whether and to what extent these transporters influence mucosal penetration or clearance of drug candidates. Moreover, data from these studies may be used as foundational inputs for future mechanistic studies and for physiologically based pharmacokinetic (PBPK) modeling of drug absorption via vaginal and intrauterine routes and support the development of both new and generic drugs. For instance, Donnelly et al. highlighted limited generic competition for drug products delivered via vaginal and intrauterine routes and discussed how PBPK models of the FRT could support product development and alternative bioequivalence approaches, as well as reduce the burden of comparative clinical endpoint bioequivalence studies in generic drug development [35].

There are several limitations associated with the current study. Although the ovary is an anatomically and physiologically relevant tissue for comprehensive gene expression profiling of the female reproductive tract, ovary samples were not available for this study, and therefore transporter and enzyme expression in this tissue could not be evaluated. Since the expression of liver transporters and metabolizing enzymes is known to be highly variable across individuals, the fold change values presented should be interpreted as relative to this specific liver reference sample rather than representation of liver population expression. Consequently, comparisons and inferences do not depend on absolute hepatic abundance, and our primary conclusions are based on within-FRT patterns (cross-tissue and cross-donor differences after reference gene normalization) and localization by IHC, which are not contingent on the liver calibrator. The FRT tissues utilized for this study were surgical waste specimens obtained through an honest broker from pre-menopausal women undergoing hysterectomy, for whom demographic information was not available to us. The lack of demographic data prevented us from exploring potential associations between gene expressions and demographics such as age, race, smoking status, co-medications, etc. In addition, the menstrual cycle phase information was not available for the tissue donors; therefore, potential menstrual cycle-dependent variation in transporter and enzyme expression could not be evaluated in our study. Gene expression of these transporters has been found to fluctuate within the menstrual cycle. For example, P-gp gene expression in the uterus was found to elevate in the proliferative endometrial stage and then reduce in the late secretory phase [36]. These limitations should be considered when interpreting these findings. Overcoming this limitation requires a prospective clinical study to recruit pre-menopausal women who will provide FRT biopsies as well as detailed demographic information. In addition, we focused on gene expression of a selected panel of drug transporters and drug-metabolizing enzymes based on their expression in the ectocervical tissue and their known effects on drug distribution in other well studied organs. From the results of our gene expression analyses, we prioritized immunohistochemical evaluations to understand the localization of three efflux drug transporters in the FRT. This information is imperative to understand how these drug transporters may play a role in the distribution of a given intravaginally delivered drug product. Quantitative analysis of protein expression and functionality study with known substrates will also be necessary to determine the roles of these drug transporters and metabolizing enzymes in drug distribution and metabolism within the FRT. These studies are beyond the scope of this manuscript.

## 5. Conclusions

In conclusion, our experimental findings expanded the gene expression profiles of drug transporters and drug-metabolizing enzymes to include the upper FRT. Our gene expression data indicated that the efflux transporters P-gp, BCRP, and MRP4 exhibited the highest expression across the ectocervix, endometrium, myometrium, and fallopian tubes, with P-gp consistently showing the greatest abundance in all evaluated FRT tissues. For drug-metabolizing enzymes, all 21 phase I and II enzymes were detectable across the FRT, and 15 were expressed at comparatively higher levels across all tissue types. These included CYP1A1, CYP1B1, CYP2B6, CYP2C8, CYP2C19, CYP3A4, UGT1A1, UGT1A3, UGT1A4, UGT1A7, UGT1A8, UGT1A10, UGT2B4, UGT2B15, and UGT2B17. P-gp, BCRP, and MRP4 proteins were observed to be localized more in the epithelial cells and stroma capillaries in the FRT. The data obtained from this investigation may provide insight to guide the selection of drug candidates and inform formulation strategies targeting the FRT across different therapeutic areas.

## Figures and Tables

**Table 1:** Summary of efflux transporter expression in human ectocervix, uterus, and fallopian tube tissues. Data are shown as the mean ± standard deviation from experiments involving 3 biological replicates from each donor. Expression values are displayed (ΔΔCt-normalized to a liver technical calibrator for plate alignment only). Interpretation should focus on relative expression within FRT tissues, not liver comparison.

	Relative mRNA Expression					
Human Tissues	P-gp	BCRP	MRP1	MRP4	MRP5	MRP7
Ectocervix	6.85 ± 9.40	1.57 ± 0.83	0.41 ± 0.22	2.39 ± 1.81	0.77 ± 0.20	0.77 ± 0.25
Endometrium	16.64 ± 17.33	8.53 ± 4.12	0.51 ± 0.24	6.93 ± 3.80	0.87 ± 0.14	0.83 ± 0.25
Myometrium	42.78 ± 22.89	26.49 ± 14.45	0.18 ± 0.08	17.91 ± 10.17	0.57 ± 0.16	0.52 ± 0.13
Fallopian Tubes	12.81 ± 5.39	7.03 ± 5.20	0.37 ± 0.17	5.88 ± 3.25	0.74 ± 0.15	0.63 ± 0.15

**Table 2:** Summary of phase I and II drug-metabolizing enzymes expression in human ectocervical, uterine, and fallopian tube tissues from six donors per tissue type. Data are shown as the mean ± standard deviation from experiments involving 3 biological replicates from each donor. Expression values are displayed (ΔΔCt-normalized to a liver technical calibrator for plate alignment only). Interpretation should focus on relative expression within FRT tissues, not liver comparison.

Human Drug Metalizing Enzymes		Relative mRNA Expression		
		Human Ectocervix	Human Uterus	Human Fallopian Tubes
Phase I	CYP1A1	0.99 ± 0.31	0.61 ± 0.14	0.57 ± 0.12
	CYP1A2	0.10 ± 0.05	0.13 ± 0.10	0.49 ± 0.22
	CYP1B1	2.43 ± 1.07	0.78 ± 0.24	1.16 ± 0.37
	CYP2B6	0.39 ± 0.22	0.62 ± 0.32	0.83 ± 0.25
	CYP2C8	0.63 ± 0.28	0.82 ± 0.21	0.91 ± 0.22
	CYP2C9	0.12 ± 0.12	0.32 ± 0.18	0.32 ± 0.12
	CYP2C19	0.67 ± 0.33	0.59 ± 0.28	0.70 ± 0.13
	CYP2D6	0.15 ± 0.11	0.27 ± 0.20	0.67 ± 0.38
	CYP2E1	0.03 ± 0.01	0.18 ± 0.14	0.36 ± 0.20
	CYP3A4	0.47 ± 0.22	0.59 ± 0.19	0.47 ± 0.16
Phase II	UGT1A1	0.85 ± 0.09	0.70 ± 0.13	0.77 ± 0.15
	UGT1A3	0.83 ± 0.21	0.68 ± 0.15	0.88 ± 0.19
	UGT1A4	0.72 ± 0.19	0.57 ± 0.11	0.74 ± 0.19
	UGT1A7	0.59 ± 0.27	0.63 ± 0.24	0.69 ± 0.13
	UGT1A8	0.72 ± 0.28	0.72 ± 0.14	0.83 ± 0.16
	UGT1A10	0.86 ± 0.18	0.84 ± 0.13	0.97 ± 0.15
	UGT2B4	0.69 ± 0.19	0.51 ± 0.13	0.44 ± 0.09
	UGT2B7	0.37 ± 0.23	0.63 ± 0.62	0.46 ± 0.63
	UGT2B15	0.85 ± 0.11	0.83 ± 0.18	0.87 ± 0.15
	UGT2B17	0.82 ± 0.80	1.08 ± 0.34	1.46 ± 0.63

## Data Availability

The authors declare that all the data supporting the findings of this study are available within the paper and its Supplemental Data.

## Supplementary Materials

The following supporting information can be downloaded at https://www.mdpi.com/article/10.3390/pharmaceutics18050629/s1, Supplementary Materials S1 includes: Figure S1. Uptake transporter expression analysis in ectocervical, endometrial, myometrial, and fallopian tube tissues (*n* = 6). Figure S2. Positive controls for each transporter and negative control without primary antibody. Figure S3. Phase I (A) and II (B) drug-metabolizing enzymes expression analysis in ectocervical, uterine, and fallopian tube tissues. Table S1. Primer sequences used for the real-time RT-qPCR of human drug transporters. Table S2. Primer sequences used for the real-time RT-qPCR of human drug-metabolizing enzymes. Table S3. (A) Summary of Ct values of GAPDH across all FRT and liver tissues used in drug transporters gene expression study. (B) Summary of Ct values of GAPDH across all FRT and liver tissues used in drug-metabolizing enzymes gene expression study. Table S4. Fold change of efflux transporter expression relative to mean FRT expression. Table S5. Summary of uptake transporter expression in human ectocervical, uterine, and fallopian tube tissues from six donors. Table S6. Fold change of uptake transporter expression relative to mean FRT expression. Table S7. Fold change of drug-metabolizing enzymes expression relative to mean FRT expression. Supplementary Materials S2 includes all the single replicate raw data used to generate the gene expression figures and tables.

## Abbreviations

The following abbreviations are used in this manuscript:

BCRP	Breast cancer resistance protein
DMEM	Dulbecco’s Modified Eagle Medium
FRT	Female reproductive tract
MRP4	Multidrug resistance-associated protein 4
P-gp	P-glycoprotein
