# Supplementary material for: Expanding the Gene Expression Profiling of Drug Transporters and Drug-Metabolizing Enzymes to Include the Upper Female Reproductive Tract
Source: Pharmaceutics. 2026 May 21;18(5):629. doi: 10.3390/pharmaceutics18050629 (PMC13210441; doi:10.3390/pharmaceutics18050629)
Supplement: Supplementary file 1 [file pharmaceutics-18-00629-s001.zip › Supp Materials s1_Revised 5.14.26_Final.pdf]

Expanding the gene expression profiling of drug transporters and drug metabolizing enzymes to include the upper female reproductive tract

An Le[1,2], Guru R. Valicherla[1,2], Junmei Zhang[2,4], Lin Wang[1,2], Mark K. Donnelly[5], Robert Bies[3] and Lisa C. Rohan[1,2,4]*

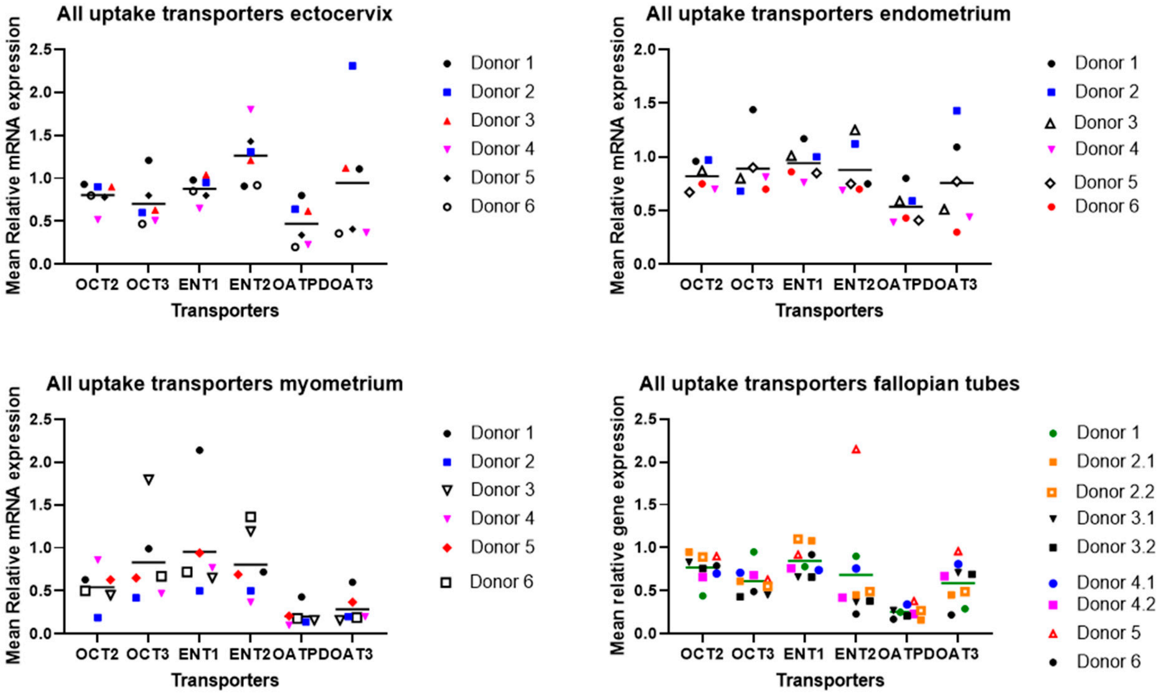

Figure S1. Uptake transporter expression analysis in ectocervical, endometrial, myometrial, and fallopian tube tissues (n=6). Data are shown as the mean from experiments involving 3 biological replicates from each donor (n=6).

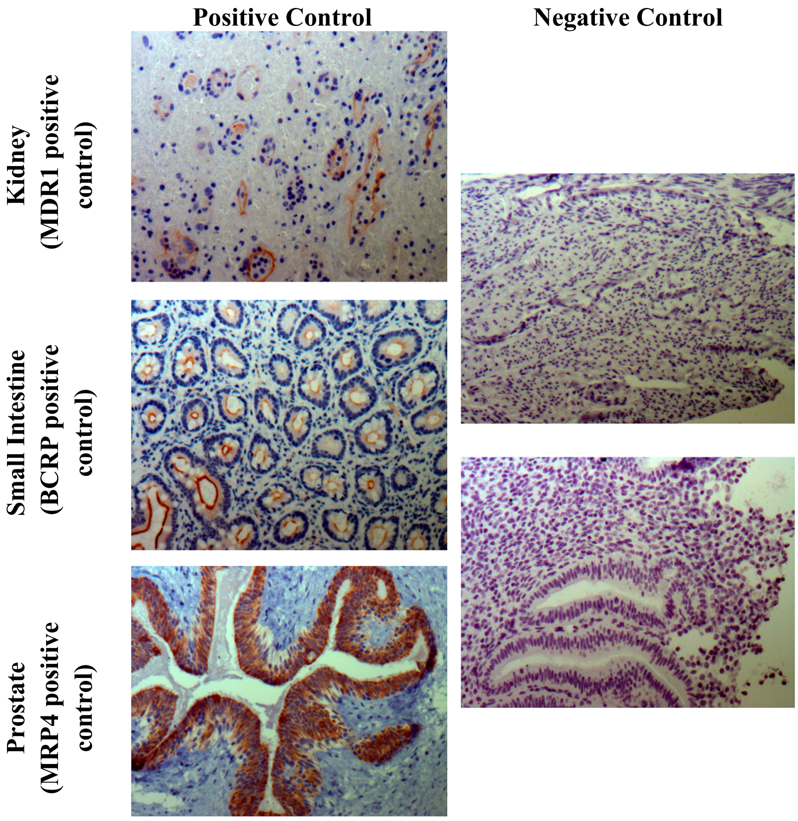

Figure S2. Positive controls for each transporter (left panel) and negative controls (right panel; top: myometrium; bottom: endometrium) without primary antibody. Images under 20x magnification were acquired using the AxioCam software, version 4.9.1 paired with the microscope, Axioskop 40.

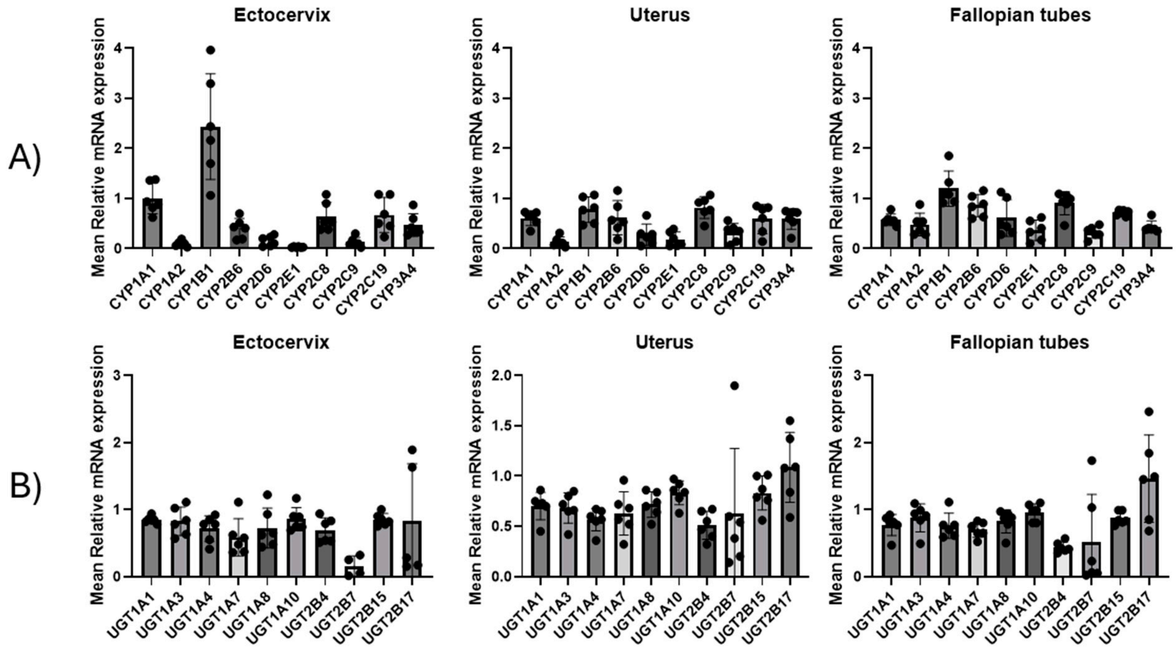

Figure S3. Expression of Phase I (A) and II (B) drug metabolizing enzymes in ectocervical, uterine, and fallopian tube tissues. Data are shown as the mean±standard deviation from experiments involving 3 biological replicates from each donor (n=6).

Table S1. Primer sequences used for the real time RT-qPCR of human drug transporters.[7]*

Common gene name (official gene symbol)	GenBank Accession no.	Primer sequence 5′ to 3′
P-gp (ABCB1)	NM_000927	Forward: CCCATCATTGCAATAGCAGG
		Reverse: TGTTCAAACTTCTGCTCCTGA
BCRP (ABCG2)	NM_004827	Forward: TGGCTGTCATGGCTTCAGTA
		Reverse: GCCACGTGATTCTTCCACAA
MRP1 (ABCC1)	NM_004996	Forward: ATGTCACGTGGAATACCAGC
		Reverse: GAAGACTGAACTCCCTTCCT
MRP4 (ABCC4)	NM_005845	Forward: ATTATTGATGAAGCGACGGC
		Reverse: GCAAAACATACGGCTCATCA
MRP5 (ABCC5)	NM_001023587	Forward: CCTTTTCACTCCCTCCATCA
		Reverse: ACAGGTCTTGGAGCTGGAGA
MRP7 (ABCC10)	NM_001198934	Forward: GCTGGTGGGCTTGTCGCTGT
		Reverse: CTGGGGTTCCTGGGGCAGGT
OCT2 (SLC22A2)	NM_003058	Forward: AGACAGTGTAGGCGCTACGA
		Reverse: GTTAAACTCGGTGACGATGGAC
OCT3 (SLC22A3)	NM_021977	Forward: ATCGTCAGCGAGTTTGACCTT
		Reverse: ACCTGTCTGCTGCATAGCCTA
ENT1 (SLC29A1)	NM_001078176	Forward: TGAGCGGAACTCTCTCAGTG
		Reverse: TGAGGTAGGTGAATAACAGCAGG
ENT2 (SLC29A2)	NM_001532	Forward: TCAGTGCAGTCCTACAGGG
		Reverse: GGCGTGATAAAGTACCCCAGG
OATP-D (SLCO3A1)	NM_001145044	Forward: GTTCCTGACCCACCAGTACAA
		Reverse: CACAGGGGTAGCACCGATG
OAT3 (SLC22A8)	NM_004254	Forward: AGCACCGTCATCTTGAATGTG
		Reverse: AGGTGTAGCAGTACCCGAGTG
GAPDH	NM_001256799	Forward-GGAGCGAGATCCCTCCAAAAT
		Reverse-GGCTGTTGTCATACTTCTCATGG

***Reference 7.** Zhou, T., et al., *Short communication: expression of transporters and metabolizing enzymes in the female lower genital tract: implications for microbicide research.* AIDS Res Hum Retroviruses, 2013. 29(11): p. 1496-503.

Table S2. Primer sequences used for the real time RT-qPCR of human drug metabolizing enzymes [7, 28]**.

Common Gene name	GenBank accession no.	Primer sequence 5′ to 3′
CYP1A1	NM_000499	Forward: TCGGCCACGGAGTTTCTTC<br>Reverse: GGTCAGCATGTGCCCAATCA
CYP1A2	NM_000761	Forward: CTTTGACAAGAACAGTGTCCG<br>Reverse: AGTGTCCAGCTCCTTCTGGAT
CYP1B1	NM_000104	Forward: AACGTCATGAGTGCCGTGTGT<br>Reverse: GGCCGGTACGTTCTCCAAATC
CYP2B6	NM_000767	Forward: GCACTCCTCACAGGACTCTTG<br>Reverse: CCCAGGTGTACCGTGAAGAC
CYP2D6	NM_000106	Forward: GCAAGAAGTCGCTGGAGCAGTG<br>Reverse: CTCACGGCTTTGTCCAAGAGAC
CYP2E1	NM_000773	Forward: GAGCACCATCAATCTCTGGACC

		Reverse: CACGGTGATACCGTCCATTGTG
CYP2C8	NM_000770	Forward: GTCCTGGTGCTGTGTCTCTC<br>Reverse: TGGTGAAAGATTTGCAGATGTCC
CYP2C9	NM_000771	Forward: ACATTGACCTTCTCCCCACCAGCC<br>Reverse: CAAATCCATTGACAACTGGAGTGG
CYP2C19	NM_000769	Forward: ACTTGGAGCTGGGACAGAGA<br>Reverse: CATCTGTGTAGGGCATGTGG
CYP3A4	NM_017460	Forward: CCGAGTGGATTTCCTTCAGCTG<br>Reverse: TGCTCGTGGTTTCATAGCCAGC
UGT1A1	NM_000463	Forward: CATGCTGGGAAGATACTGTTGAT<br>Reverse: GCCCGAGACTAACAAAAGACTCT
UGT1A3	NM_019093	Forward: ATGGCAATGTTGAACAATATG<br>Reverse: GGTCTGAATTGGTTGTTAGTAATC
UGT1A4	NM_007120	Forward: ACGCTGGGCTACACTCAAGG<br>Reverse: GACAGGTACTTAGCCAGCACC
UGT1A7	NM_019077	Forward: TGGCTCGTGCAGGGTGGACTG<br>Reverse: TTCGCAATGGTGCCGTCCAGC
UGT1A8	NM_019076	Forward: CTGCTGACCTGTGGCTTTGCT<br>Reverse: CCATTGAGCATCGGCGAAAT
UGT1A9	NM_021027	Forward: GAGGAACATTTATTATGCCACCG<br>Reverse: CCATTGATCCCAAAGAGAAAACC
UGT1A10	NM_019075	Forward: CCTCTTTCCTATGTCCCCAATGA<br>Reverse: GCAACAACCAAATTGATGTGTG
UGT2B4	NM_021139	Forward: TCTACTCTTAAATTTGAAGTTTATCCTGT<br>Reverse: CTCATAGATGCCATTGGCTCCAC
UGT2B7	NM_001074	Forward:<br>GACGTATGGCTTATTCGAAACTCCTGGAATTTTCAG<br>Reverse:<br>GCAATGTTATCAGGTTGATCGCAAACAATGGAATC
UGT2B15	NM_001076	Forward: GTGTTGGGAATATTATGACTACAGTAAC<br>Reverse: CGCCTCATAGATGCCATTGGTTC
UGT2B17	NM_001077	Forward: GTGTTGGGAATATTCTGACTATAATATA<br>Reverse: CAGGTACATAGGAAGGAGGGAA

***Reference 7.** Zhou, T., et al., *Short communication: expression of transporters and metabolizing enzymes in the female lower genital tract: implications for microbicide research.* AIDS Res Hum Retroviruses, 2013. 29(11): p. 1496-503.

****Reference 28.** Valicherla, G.R., et al., *Investigating the contribution of drug-metabolizing enzymes in drug-drug interactions of dapivirine and miconazole.* Pharmaceutics, 2021. 13(12).

Table S3. A) Summary of Ct values of GAPDH across all FRT and liver tissues used in drug transporters gene expression study. B) Summary of Ct values of GAPDH across all FRT and liver tissues used in drug metabolizing enzymes gene expression study.

A)

	Liver 1	Liver 2	Ectocervix (n=6)	Endometrium (n=6)	Myometrium (n=6)	Fallopian Tubes (n=6)
Average GAPDH Ct	23.68	23.49	22.35	22.68	22.50	21.51
Standard deviation GAPDH Ct	0.21	0.35	0.63	0.72	0.84	0.56

B)

	Liver	Ectocervix (n=6)	Uterus (n=6)	Fallopian Tubes (n=6)
Average GAPDH Ct	23.97	21.71	21.36	20.81
Standard deviation GAPDH Ct	0.95	0.32	0.69	0.63

Table S4. Fold change of efflux transporter expression relative to mean FRT expression. Values represent fold change relative to the mean expression across all FRT tissues for each efflux transporter. The fold change is provided for visualization only and cannot reflect the variability in gene expression which is represented in the original table (Table 1).

	Fold change relative to mean FRT expression					
Human Tissues	P-gp	BCRP	MRP1	MRP4	MRP5	MRP7
Ectocervix	0.35	0.14	1.11	0.29	1.04	1.12
Endometrium	0.84	0.78	1.39	0.84	1.18	1.21
Myometrium	2.16	2.43	0.49	2.16	0.77	0.76
Fallopian Tubes	0.65	0.64	1.00	0.71	1.00	0.92

Table S5. Summary of uptake transporter expression in human ectocervical, uterine, and fallopian tube tissues from six donors. Data are shown as the mean±standard deviation from experiments involving 3 biological replicates from each donor (n=6). Expression values (ΔΔCt-normalized to a liver technical calibrator for plate alignment only). Interpretation should focus on relative expression within FRT tissues, not liver comparison.

	Relative mRNA expression					
Human Tissues	OCT2	OCT3	ENT1	ENT2	OATPD	OAT3
Ectocervix	0.81±0.18	0.70±0.28	0.88±0.16	1.26±0.45	0.47±0.25	0.96±0.76
Endometrium	0.82±0.16	0.89±0.34	0.94±0.17	0.88±0.31	0.53±0.17	0.76±0.41

Myometrium	0.54±0.22	0.83±0.49	0.96±0.59	0.8±0.42	0.2±0.12	0.29±0.19
Fallopian Tubes	0.77±0.16	0.61±0.16	0.84±0.17	0.68±0.57	0.25±0.09	0.59±0.25

Table S6. Fold change of uptake transporter expression relative to mean FRT expression. Values represent fold change relative to the mean expression across all FRT tissues for each uptake transporter. The fold change is provided for visualization only and cannot reflect the variability in gene expression which is represented in the original table (Table S5).

	Fold change relative to mean FRT expression					
Human Tissues	OCT2	OCT3	ENT1	ENT2	OATPD	OAT3
Ectocervix	1.10	0.92	0.97	1.39	1.30	1.48
Endometrium	1.12	1.17	1.04	0.97	1.46	1.17
Myometrium	0.73	1.09	1.06	0.88	0.55	0.45
Fallopian Tubes	1.05	0.81	0.93	0.75	0.69	0.91

Table S7. Fold change of drug metabolizing enzymes expression relative to mean FRT expression. Values represent fold change relative to the mean expression across all FRT tissues for each drug metabolizing enzyme. The fold change is provided for visualization only and cannot reflect the variability in gene expression which is represented in the original table (Table 2).

Human drug metalizing enzymes		Relative mRNA expression		
		Human Ectocervix	Human Uterus	Human Fallopian Tubes
Phase I	CYP1A1	1.37	0.84	0.79
	CYP1A2	0.42	0.54	2.04
	CYP1B1	1.67	0.54	0.80
	CYP2B6	0.63	1.01	1.35
	CYP2C8	0.80	1.04	1.16
	CYP2C9	0.47	1.26	1.26
	CYP2C19	1.03	0.90	1.07
	CYP2D6	0.41	0.74	1.84
	CYP2E1	0.16	0.95	1.89
	CYP3A4	0.92	1.16	0.92
Phase II	UGT1A1	1.10	0.91	1.00
	UGT1A3	1.04	0.85	1.10
	UGT1A4	1.06	0.84	1.09
	UGT1A7	0.93	0.99	1.08
	UGT1A8	0.95	0.95	1.10
	UGT1A10	0.97	0.94	1.09
	UGT2B4	1.26	0.93	0.80
	UGT2B7	0.76	1.29	0.95
	UGT2B15	1.00	0.98	1.02
	UGT2B17	0.73	0.96	1.30
