# Supplementary material for: Expanding the Gene Expression Profiling of Drug Transporters and Drug-Metabolizing Enzymes to Include the Upper Female Reproductive Tract
Source: Pharmaceutics. 2026 May 21;18(5):629. doi: 10.3390/pharmaceutics18050629 (PMC13210441; doi:10.3390/pharmaceutics18050629)
Supplement: Supplementary file 1 [file pharmaceutics-18-00629-s001.zip › Supp Materials s2_Revised 5.14.26_Final.pdf]

Expanding the gene expression profiling of drug transporters and drug metabolizing enzymes to include the upper female reproductive tract

An Le[1,2], Guru R. Valicherla[1,2], Junmei Zhang[2,4], Lin Wang[1,2], Mark K. Donnelly[5], Robert Bies[3] and Lisa C. Rohan[1,2,4]*

The following raw data obtained from qPCR is used to generate Tables 1, S4- S6, and Figures 1-2, and S1. Only donors 4, 5, and 6 in the ectocervix, endometrium, and myometrium of drug transporters are matched donors. The color-coded raw data represents matched donors, which are shown in Figure 2 and Figure S1.

Ectocervix – Efflux Transporters

	**Pgp**	**BCRP**	**MRP1**	**MRP4**	**MRP5**	**MRP7**
Three separate donors with 3 replicates for each gene.	2.29	0.85	0.32	2.40	0.57	0.50
	2.11	0.97	0.25	2.29	0.51	0.47
	2.69	0.78	0.22	2.57	0.45	0.46
	2.88	2.12	0.34	1.71	0.83	0.70
	3.18	1.69	0.32	3.71	0.59	0.55
	3.92	2.15	0.28	1.39	0.59	0.50
	31.86	3.59	0.29	7.44	0.78	0.78
	21.83	2.02	0.20	5.60	0.48	0.55
	26.48	2.91	0.20	4.59	0.69	0.64
Donor 4	**1.14**	**0.73**	0.42	**1.15**	0.92	0.81
	**0.87**	**0.34**	0.32	**1.17**	0.84	0.82
	**1.14**	**0.67**	0.36	**1.14**	0.78	0.78
Donor 5	**2.90**	**1.54**	0.98	**1.87**	1.07	1.21
	**2.21**	**1.73**	0.68	**1.12**	0.76	0.98
	**2.39**	**1.91**	0.33	**1.27**	1.16	1.22
Donor 6	**4.98**	**1.57**	0.75	**1.29**	0.93	0.90
	**4.70**	**1.14**	0.46	**1.13**	0.86	0.85
	**5.74**	**1.57**	0.65	**1.16**	1.00	1.11
**Average**	6.85	1.57	0.41	2.39	0.77	0.77
**Standard deviation**	9.40	0.83	0.22	1.81	0.20	0.25

Endometrium – Efflux Transporters

	**Pgp**	**BCRP**	**MRP1**	**MRP4**	**MRP5**	**MRP7**
Three separate donors with 3 replicates for each gene.	9.32	5.85	0.29	4.34	0.80	0.62
	9.74	5.33	0.21	4.05	0.68	0.57
	9.90	5.80	0.29	4.99	0.78	0.60
	16.45	16.44	0.24	11.68	0.79	0.56
	16.61	16.64	0.23	12.82	0.72	0.70
	18.44	18.25	0.28	14.46	0.74	0.71
	52.72	8.37	0.49	9.98	0.76	0.62
	50.51	8.36	0.37	9.90	0.76	0.62

	55.68	9.32	0.49	11.75	0.81	0.65
Donor 4	**8.73**	**6.46**	0.57	**6.42**	1.01	0.88
	**7.45**	**7.19**	0.44	**6.85**	1.01	0.91
	**9.57**	**6.82**	0.51	**5.89**	0.95	0.84
Donor 5	**1.88**	**5.89**	0.99	**4.21**	0.90	1.04
	**2.33**	**5.71**	0.86	**3.66**	0.93	0.99
	**2.64**	**7.23**	0.86	**2.74**	0.99	1.12
Donor 6	**10.39**	**7.82**	0.65	**4.93**	1.22	1.41
	**9.13**	**6.69**	0.80	**3.75**	1.05	1.15
	**8.07**	**5.35**	0.67	**2.35**	0.84	1.01
**Average**	16.64	8.53	0.51	6.93	0.87	0.83
**Standard deviation**	17.33	4.12	0.24	3.80	0.14	0.25

Myometrium – Efflux Transporters

	**Pgp**	**BCRP**	**MRP1**	**MRP4**	**MRP5**	**MRP7**
Three separate donors with 3 replicates for each gene.	5.69	8.79	0.18	3.84	0.45	0.39
	6.35	8.66	0.22	4.25	0.50	0.39
	5.61	9.45	0.19	3.28	0.51	0.39
	36.09	43.69	0.17	24.55	0.71	0.49
	32.95	40.97	0.14	20.82	0.71	0.53
	35.10	43.52	0.18	25.46	0.76	0.55
	69.66	11.02	0.20	13.74	0.68	0.54
	66.87	8.66	0.20	11.28	0.54	0.45
	61.46	8.88	0.23	10.41	0.54	0.45
Donor 4	**24.62**	**31.11**	0.09	**20.65**	0.65	0.38
	**31.14**	**27.40**	0.10	**24.29**	0.67	0.44
	**26.70**	**32.76**	0.08	**16.86**	0.78	0.38
Donor 5	**65.73**	**43.94**	0.15	**35.45**	0.25	0.68
	**65.19**	**43.22**	0.12	**33.99**	0.29	0.77
	**53.94**	**43.43**	0.14	**33.76**	0.29	0.78
Donor 6	**63.36**	**22.07**	0.39	**12.81**	0.68	0.59
	**53.34**	**23.20**	0.26	**12.41**	0.63	0.57
	**66.34**	**26.12**	0.27	**14.60**	0.66	0.61
**Average**	42.78	26.49	0.18	17.91	0.57	0.52
**Standard deviation**	22.89	14.45	0.08	10.17	0.16	0.13

Fallopian Tubes – Efflux Transporters

	P-gp	BCRP	MRP1	MRP4	MRP5	MRP7
Six separate donors	24.14	10.00	0.44	12.14	0.86	0.78
	22.85	10.66	0.45	12.34	0.79	0.78
	19.95	10.16	0.47	10.96	0.75	0.74

with 3 replicates for each gene. Some donors provided 2 different tubes (left and right)	13.64	5.38	0.17	7.94	0.34	0.31
	14.85	5.84	0.17	8.54	0.40	0.31
	14.80	5.80	0.19	8.64	0.39	0.14
	13.89	3.25	0.26	7.69	0.76	0.58
	13.53	3.85	0.32	8.95	0.77	0.54
	17.68	4.16	0.23	10.51	0.66	0.62
	17.85	3.92	0.33	6.43	0.75	0.78
	20.13	4.28	0.35	7.52	0.73	0.65
	18.60	4.29	0.52	8.01	0.62	0.61
	5.43	1.84	0.69	3.18	0.83	0.79
	4.51	2.17	0.68	2.75	0.97	0.70
	6.13	1.92	0.71	2.83	0.97	0.64
	7.92	2.60	0.55	3.75	0.84	0.70
	7.39	2.42	0.58	4.64	0.80	0.63
	6.71	2.21	0.53	4.21	0.76	0.72
	12.95	9.00	0.34	3.32	0.70	0.64
	12.88	7.91	0.36	3.07	0.80	0.70
	11.78	8.63	0.36	2.96	0.73	0.72
	11.74	8.70	0.21	3.35	0.73	0.64
	10.64	6.33	0.27	3.29	0.92	0.65
	8.57	6.65	0.27	2.58	0.77	0.67
	9.30	18.73	0.21	2.73	0.77	0.65
	7.73	17.58	0.22	3.15	0.71	0.74
	10.24	21.43	0.22	3.19	0.81	0.69
**Average**	12.81	7.03	0.37	5.88	0.74	0.63
**Standard deviation**	5.39	5.20	0.17	3.25	0.15	0.15

Ectocervix – Uptake Transporters

	OCT 2	OCT 3	ENT1	ENT2	OATP-D	OAT 3
Six separate donors with 3 replicates for each gene.	1.03	1.06	1.12	0.85	0.82	1.11
	0.87	1.12	0.95	0.90	0.81	1.30
	0.90	1.43	0.86	1.00	0.77	0.93
	0.92	0.62	1.04	1.28	0.94	2.50
	0.66	0.60	0.78	0.95	0.41	1.47
	1.13	0.60	1.03	1.70	0.57	2.95
	0.89	0.70	1.11	1.11	0.71	0.99
	0.79	0.54	0.91	1.06	0.55	0.94
	1.03	0.66	1.09	1.47	0.61	1.42
	0.50	0.43	0.62	1.08	0.23	0.29
	0.51	0.53	0.63	2.42	0.27	0.50

	0.55	0.56	0.70	1.90	0.20	0.32
	0.76	1.01	0.89	1.07	0.28	0.23
	0.84	0.74	0.72	1.84	0.35	0.50
	0.75	0.64	0.79	1.38	0.38	0.48
	0.84	0.63	0.92	1.13	0.28	0.66
	0.71	0.33	0.77	1.00	0.18	0.29
	0.85	0.46	0.85	0.63	0.15	0.42
**Average**	0.81	0.70	0.88	1.26	0.47	0.96
**Standard deviation**	0.18	0.28	0.16	0.45	0.25	0.76

Endometrium – Uptake Transporters

	OCT 2	OCT 3	ENT1	ENT2	OATP-D	OAT 3
Six separate donors with 3 replicates for each gene.	1.07	1.98	1.38	0.95	0.78	1.22
	0.99	0.76	1.16	0.57	0.71	0.95
	0.83	1.57	0.97	0.73	0.91	1.09
	0.68	0.76	0.68	0.61	0.37	0.44
	0.67	0.80	0.75	0.60	0.43	0.28
	0.75	0.88	0.86	0.87	0.35	0.59
	0.80	0.91	0.75	0.76	0.37	0.88
	0.50	0.87	0.84	0.66	0.41	0.78
	0.72	0.93	0.97	0.82	0.46	0.66
	1.13	0.61	1.11	1.00	0.70	1.48
	0.78	0.69	0.94	1.14	0.59	1.44
	1.00	0.74	0.96	1.23	0.48	1.39
	0.75	0.73	0.82	0.70	0.39	0.34
	0.70	0.55	0.85	0.82	0.41	0.27
	0.81	0.85	0.88	0.59	0.45	0.33
	0.91	0.83	1.05	0.80	0.53	0.55
	0.77	0.80	0.94	1.10	0.76	0.53
	0.93	0.77	1.03	1.85	0.47	0.45
**Average**	0.82	0.89	0.94	0.88	0.53	0.76
**Standard deviation**	0.16	0.34	0.17	0.31	0.17	0.41

Myometrium – Uptake Transporters

	OCT 2	OCT 3	ENT1	ENT2	OATP-D	OAT 3
Six separate donors with 3 replicates	0.77	0.89	2.65	0.81	0.49	0.79
	0.44	0.88	1.67	0.59	0.36	0.38
	0.68	1.21	2.11	0.76	0.44	0.63
	0.90	0.41	0.75	0.42	0.08	0.26
	0.89	0.44	0.79	0.32	0.05	0.11

for each gene.	0.78	0.56	0.78	0.36	0.16	0.23
	0.64	0.66	0.95	0.54	0.20	0.32
	0.64	0.59	0.93	0.82	0.20	0.44
	0.61	0.70	0.94	0.71	0.24	0.34
	0.25	0.39	0.61	0.44	0.14	0.33
	0.18	0.50	0.43	0.60	0.14	0.15
	0.14	0.36	0.45	0.45	0.15	0.10
	0.56	0.74	0.74	1.28	0.18	0.15
	0.46	0.66	0.69	1.37	0.18	0.26
	0.47	0.60	0.73	1.43	0.18	0.15
	0.50	1.74	0.62	1.80	0.18	0.15
	0.42	1.88	0.68	0.70	0.18	0.17
	0.44	1.75	0.66	1.07	0.10	
**Average**	0.54	0.83	0.96	0.80	0.20	0.29
**Standard deviation**	0.22	0.49	0.59	0.42	0.12	0.19

Fallopian Tubes – Uptake Transporters

	OCT 2	OCT 3	ENT1	ENT2	OATP-D	OAT 3
Six separate donors with 3 replicates for each gene. Some donors provided 2 different tubes (left and right)	0.86	0.55	0.96	0.28	0.20	0.29
	0.71	0.52	0.89	0.22	0.19	0.23
	0.79	0.41	0.91	0.20	0.13	0.14
	0.42	0.86	0.70	0.90	0.11	0.30
	0.50	1.09	0.85	0.92	0.43	0.48
	0.40	0.89	0.78	0.87	0.20	0.11
	0.98	0.66	1.18	0.41	0.20	0.46
	0.94	0.54	1.01	0.55	0.13	0.48
	0.91	0.62	1.04	0.38	0.16	0.39
	0.93	0.50	1.13	0.40	0.19	0.53
	0.88	0.62	1.10	0.45	0.22	0.46
	0.87	0.54	1.07	0.61	0.40	0.82
	0.91	0.45	0.69	0.38	0.23	0.76
	0.79	0.43	0.67	0.33	0.31	0.55
	0.80	0.46	0.62	0.41	0.26	0.59
	0.74	0.41	0.63	0.36	0.17	0.74
	0.81	0.39	0.68	0.38	0.25	0.72
	0.73	0.48	0.66	0.42	0.20	0.77
	0.70	0.66	0.73	0.75	0.31	0.80
	0.71	0.72	0.77	0.71	0.36	0.85
	0.68	0.73	0.71	0.82	0.35	0.62
	0.64	0.66	0.70	0.38	0.24	0.66
	0.71	0.70	0.79	0.50	0.26	0.73

	0.65	0.67	0.78	0.39	0.20	0.88
	0.86	0.59	0.85	2.09	0.39	1.07
	0.88	0.60	0.91	2.02	0.37	0.94
	0.97	0.70	1.00	2.35	0.38	
**Average**	0.77	0.61	0.84	0.68	0.25	0.59
**Standard deviation**	0.16	0.16	0.17	0.57	0.09	0.25

The following phase I and II drug metabolizing enzymes raw data obtained from qPCR is used to generate Tables 2, S7, and Figure S3. For all 3 tissue types, 6 donors were included. For the fallopian tubes, some donors provided 2 tubes (left and right).

CYP1A1

	Cervix	Uterine	FT
Six separate donors with 3 replicates for each gene. Some fallopian tubes donors provided 2 different tubes (left and right)	0.63	0.57	0.39
	0.60	0.51	0.45
	0.62	0.52	0.47
	0.82	0.36	0.57
	0.86	0.33	0.63
	0.78	0.34	0.67
	1.00	0.75	0.54
	0.71	0.64	0.39
	1.12	0.78	0.47
	1.18	0.72	0.56
	1.61	0.75	0.63
	1.29	0.71	0.59
	0.87	0.67	0.58
	0.90	0.67	0.51
	0.86	0.65	0.51
	1.55	0.70	0.64
	1.14	0.59	0.52
	1.47	0.67	0.51
			0.84
			0.62
			0.87
**Average**	0.99	0.61	0.57
**Standard deviation**	0.32	0.14	0.12

CYP1A2

	**Cervix**	**Uterine**	**FT**

Six separate donors with 3 replicates for each gene. Some fallopian tubes donors provided 2 different tubes (left and right)	0.13	0.07	0.46
	0.11	0.09	0.67
	0.11	0.07	0.50
	0.06	0.03	1.14
	0.05	0.03	0.66
	0.07	0.03	0.86
	0.18	0.18	0.53
	0.19	0.18	0.52
	0.20	0.18	0.54
	0.03	0.30	0.57
	0.03	0.32	0.53
	0.04	0.31	0.56
	0.15	0.11	0.32
	0.09	0.11	0.26
	0.07	0.09	0.26
	0.09	0.08	0.36
	0.07	0.08	0.39
	0.07		0.36
			0.26
			0.29
			0.30
**Average**	0.10	0.13	0.49
**Standard deviation**	0.05	0.10	0.22

CYP1B1

	**Cervix**	**Uterine**	**FT**
Six separate donors with 3 replicates for each gene. Some fallopian tubes donors provided 2 different tubes (left and right)	0.94	0.57	0.72
	0.99	0.48	1.16
	1.23	0.45	0.85
	1.50	0.54	0.98
	1.69	0.37	1.04
	1.92	0.51	1.07
	2.41	0.86	1.08
	2.15	0.74	0.69
	2.75	0.72	0.76
	3.38	0.70	1.77
	3.91	0.82	1.78
	4.60	0.94	2.01
	2.65	1.09	1.03
	3.31	1.08	0.98

	3.93	1.04	1.25
	2.84	1.03	1.69
	2.18	0.98	1.28
	1.46	1.09	0.99
			0.92
			1.30
			1.02
**Average**	2.44	0.78	1.16
**Standard deviation**	1.07	0.24	0.37

CYP2B6

	**Cervix**	**Uterine**	**FT**
Six separate donors with 3 replicates for each gene. Some fallopian tubes donors provided 2 different tubes (left and right)	0.44	0.78	0.64
	0.46	0.91	0.6
	0.40	0.82	0.56
	0.08	1.15	0.65
	0.14	1.12	0.76
	0.06	1.22	0.76
	0.14	0.47	0.55
	0.27	0.48	0.39
	0.12	0.31	0.64
	0.49	0.58	0.92
	0.54	0.54	0.87
	0.52	0.59	0.89
	0.37	0.50	0.91
	0.45	0.48	0.64
	0.40	0.62	0.96
	0.58	0.19	1.38
	0.82	0.16	0.84
	0.70	0.20	0.91
			1.07
			1.37
			1.05
**Average**	0.39	0.62	0.83
**Standard deviation**	0.22	0.32	0.25

CYP2D6

	**Cervix**	**Uterine**	**FT**
Six separate donors	0.08	0.10	0.34
	0.10	0.11	0.25
	0.11	0.13	0.28

with 3 replicates for each gene. Some fallopian tubes donors provided 2 different tubes (left and right)	0.04	0.05	0.32
	0.04	0.05	0.30
	0.05	0.06	0.36
	0.18	0.31	0.45
	0.20	0.23	0.40
	0.21	0.36	0.46
	0.06	0.68	1.26
	0.02	0.75	1.23
	0.08	0.56	0.90
	0.27	0.23	0.31
	0.27	0.23	0.71
	0.16	0.24	0.41
	0.43	0.31	0.97
	0.25	0.29	1.06
	0.16	0.27	1.22
			0.70
			0.83
			1.38
**Average**	0.15	0.28	0.67
**Standard deviation**	0.11	0.20	0.38

CYP2E1

	Cervix	Uterine	FT
Six separate donors with 3 replicates for each gene. Some fallopian tubes donors provided 2 different tubes (left and right)	0.04	0.11	0.68
	0.04	0.07	0.55
	0.04	0.06	0.63
	0.03	0.41	0.09
	0.03	0.36	0.09
	0.02	0.39	0.13
	0.03	0.20	0.41
	0.03	0.23	0.41
	0.04	0.18	0.42
	0.02	0.06	0.64
	0.02	0.06	0.53
	0.02	0.05	0.48
	0.03	0.33	0.26
	0.03	0.34	0.00
	0.02	0.32	0.25
	0.02	0.05	0.07
	0.02	0.05	0.37
	0.02	0.05	0.37

			0.32
			0.33
			0.50
**Average**	0.03	0.18	0.36
**Standard deviation**	0.01	0.14	0.20

CYP2C8

	**Cervix**	**Uterine**	**FT**
Six separate donors with 3 replicates for each gene. Some fallopian tubes donors provided 2 different tubes (left and right)	0.55	0.71	0.87
	0.46	0.75	0.90
	0.58	0.75	0.89
	0.41	0.48	0.85
	0.35	0.49	0.98
	0.39	0.37	0.88
	0.55	0.80	0.88
	0.52	0.79	0.82
	0.50	0.71	1.23
	0.44	0.96	1.01
	0.37	0.90	1.11
	0.34	0.90	1.06
	0.90	0.98	1.02
	0.88	0.88	1.15
	0.91	1.02	1.10
	1.25	1.17	1.20
	0.99	1.02	0.87
	1.00	1.01	0.92
			0.42
			0.50
			0.45
**Average**	0.63	0.82	0.91
**Standard deviation**	0.28	0.21	0.22

CYP2C9

	Cervix	Uterine	FT
Six separate donors with 3 replicates for each gene.	0.18	0.16	0.44
	0.17	0.08	0.62
	0.17	0.17	0.39
	0.09	0.07	0.51
	0.09	0.07	0.37
	0.12	0.06	0.34

Some fallopian tubes donors provided 2 different tubes (left and right)	0.08	0.37	0.25
	0.08	0.33	0.28
	0.10	0.46	0.30
	0.08	0.61	0.34
	0.07	0.52	0.41
	0.08	0.56	0.37
	0.01	0.35	0.13
	0.01	0.33	0.17
	0.03	0.38	0.13
	0.36	0.43	0.33
	0.28	0.42	0.34
	0.20	0.38	0.35
			0.22
			0.19
			0.27
**Average**	0.12	0.32	0.32
**Standard deviation**	0.12	0.18	0.12

CYP2C19

	**Cervix**	**Uterine**	**FT**
Six separate donors with 3 replicates for each gene. Some fallopian tubes donors provided 2 different tubes (left and right)	0.69	0.58	0.60
	0.67	0.63	0.81
	0.73	0.60	0.60
	1.08	0.74	0.72
	1.05	0.83	0.72
	1.12	0.78	0.66
	0.49	0.85	0.53
	0.45	0.88	0.44
	0.40	0.73	0.71
	0.22	0.75	0.93
	0.25	0.87	0.69
	0.22	0.93	0.65
	0.52	0.37	0.78
	0.48	0.30	0.69
	0.50	0.34	0.75
	1.22	0.13	0.93
	1.03	0.14	0.68
	0.99	0.14	0.72
			0.55
			0.93
			0.69

**Average**	0.67	0.59	0.70
**Standard deviation**	0.33	0.28	0.13

CYP3A4

	**Cervix**	**Uterine**	**FT**
Six separate donors with 3 replicates for each gene. Some fallopian tubes donors provided 2 different tubes (left and right)	0.58	0.79	0.42
	0.58	0.72	0.39
	0.63	0.67	0.37
	0.29	0.65	0.41
	0.35	0.60	0.37
	0.36	0.54	0.37
	0.26	0.52	0.39
	0.24	0.44	0.41
	0.30	0.62	0.38
	0.55	0.23	0.41
	0.35	0.23	0.42
	0.36	0.16	0.40
	0.35	0.69	0.35
	0.35	0.70	0.36
	0.28	0.80	0.33
	1.00	0.79	0.94
	0.81	0.74	0.68
	0.79	0.71	0.85
			0.47
			0.44
			0.63
**Average**	0.47	0.59	0.47
**Standard deviation**	0.22	0.19	0.16

UGT1A1

	**Cervix**	**Uterine**	**FT**
Six separate donors with 3 replicates for each gene. Some fallopian tubes donors provided 2	0.73	0.82	0.71
	0.78	0.70	0.92
	0.82	0.74	0.80
	0.67	0.39	0.71
	0.79	0.45	0.79
	0.99	0.52	0.77
	0.88	0.70	0.73
	0.80	0.67	0.70
	0.85	0.73	0.89
	0.86	0.78	0.79

different tubes (left and right)	0.85	0.73	0.87
	0.82	0.68	0.94
	0.84	0.73	0.85
	0.84	0.74	0.82
	0.91	0.69	0.71
	1.01	0.87	1.01
	0.83	0.90	0.87
	0.99	0.82	0.88
			0.43
			0.46
			0.52
**Average**	0.85	0.70	0.77
**Standard deviation**	0.09	0.13	0.15

UGT1A3

	Cervix	Uterine	FT
Six separate donors with 3 replicates for each gene. Some fallopian tubes donors provided 2 different tubes (left and right)	1.02	0.83	1.12
	0.97	0.79	0.89
	1.07	0.78	0.96
	0.91	0.47	0.48
	0.77	0.45	0.54
	0.83	0.32	0.44
	0.92	0.66	1.07
	0.76	0.64	1.12
	0.66	0.65	1.07
	0.63	0.82	0.89
	0.61	0.66	0.96
	0.48	0.68	0.94
	0.68	0.61	0.94
	0.70	0.69	0.79
	0.50	0.67	0.93
	1.08	0.91	0.97
	1.06	0.84	0.94
	1.20	0.79	0.92
			0.88
			0.83
			0.80
**Average**	0.83	0.68	0.88
**Standard deviation**	0.21	0.15	0.19

UGT1A4

	Cervix	Uterine	FT
Six separate donors with 3 replicates for each gene. Some fallopian tubes donors provided 2 different tubes (left and right)	0.60	0.57	0.75
	0.58	0.54	0.82
	0.47	0.53	0.75
	0.45	0.67	0.73
	0.40	0.61	0.70
	0.38	0.66	0.68
	0.95	0.35	0.57
	0.78	0.34	0.60
	0.83	0.40	0.58
	0.81	0.65	0.63
	0.84	0.60	0.69
	0.70	0.54	0.67
	0.81	0.66	0.62
	0.73	0.66	0.48
	0.79	0.69	0.59
	1.05	0.60	1.34
	0.80	0.49	0.94
	0.90	0.62	1.06
			0.91
			0.77
			0.61
**Average**	0.72	0.57	0.74
**Standard deviation**	0.19	0.11	0.19

UGT1A7

	Cervix	Uterine	FT
Six separate donors with 3 replicates for each gene. Some fallopian tubes donors provided 2 different tubes (left and right)	0.38	0.71	0.64
	0.43	0.72	0.71
	0.63	0.63	0.61
	0.42	1.33	0.65
	0.34	0.80	0.59
	0.32	0.76	0.70
	0.59	0.57	0.65
	0.54	0.50	0.53
	0.62	0.64	0.76
	0.43	0.54	0.73
	0.34	0.45	0.85
	0.34	0.56	0.90
	0.55	0.76	0.89
	0.70	0.72	0.78

	0.60	0.68	0.74
	1.09	0.30	0.38
	1.09	0.27	0.54
	1.15	0.38	0.63
			0.79
			0.77
			0.69
**Average**	0.59	0.63	0.69
**Standard deviation**	0.27	0.24	0.13

UGT1A8

	Cervix	Uterine	FT
Six separate donors with 3 replicates for each gene. Some fallopian tubes donors provided 2 different tubes (left and right)	0.95	0.96	1.00
	0.95	0.84	0.97
	0.88	0.80	0.94
	0.63	0.69	0.84
	0.64	0.65	0.87
	0.60	0.59	0.92
	0.72	0.58	0.90
	0.62	0.80	0.77
	0.60	0.70	0.96
	0.50	0.78	0.86
	0.53	0.62	0.92
	0.40	0.81	1.03
	0.40	0.91	0.89
	0.42	0.78	0.87
	0.51	0.82	0.84
	1.25	0.51	0.42
	1.17	0.46	0.52
	1.24	0.58	0.55
			0.91
			0.79
			0.63
**Average**	0.72	0.72	0.83
**Standard deviation**	0.28	0.14	0.16

UGT1A10

	Cervix	Uterine	FT
Six separate	0.83	0.98	1.07
	0.87	0.85	1.25

donors with 3 replicates for each gene. Some fallopian tubes donors provided 2 different tubes (left and right)	0.97	0.94	0.98
	0.65	0.83	0.94
	0.77	0.88	1.06
	0.80	0.81	1.14
	0.84	0.78	0.96
	0.87	0.86	1.06
	1.00	0.95	1.22
	0.76	0.78	0.80
	0.71	0.70	1.07
	0.61	0.87	1.08
	0.75	1.04	1.03
	0.81	1.00	1.00
	0.68	0.88	0.94
	1.18	0.57	0.85
	1.10	0.57	0.83
	1.24	0.75	0.72
			0.71
			0.94
			0.75
**Average**	0.86	0.84	0.97
**Standard deviation**	0.18	0.13	0.15

UGT2B4

	Cervix	Uterine	FT
Six separate donors with 3 replicates for each gene. Some fallopian tubes donors provided 2 different tubes (left and right)	0.51	0.74	0.58
	0.61	0.64	0.53
	0.50	0.62	0.58
	0.59	0.63	0.46
	0.54	0.58	0.43
	0.47	0.73	0.57
	0.99	0.57	0.33
	0.79	0.49	0.29
	1.05	0.60	0.44
	0.46	0.41	0.48
	0.54	0.38	0.39
	0.54	0.41	0.40
	0.85	0.47	0.39
	0.75	0.48	0.41
	0.76	0.46	0.36
	0.70	0.34	0.37
	1.00	0.30	0.50

	0.80	0.33	0.37
**Average**	0.69	0.51	0.44
**Standard deviation**	0.19	0.13	0.09

UGT2B7

	Cervix	Uterine	FT
Six separate donors with 3 replicates for each gene. Some fallopian tubes donors provided 2 different tubes (left and right)	0.25	0.54	1.04
	0.28	0.47	0.99
	0.22	0.62	1.06
	0.24	0.55	0.08
	0.40	0.69	0.09
	0.33	0.56	0.09
	0.57	1.77	0.06
	0.55	1.73	0.03
	0.47	2.20	0.03
	0.15	0.26	0.04
	0.11	0.25	0.05
	0.09	0.11	0.06
	0.89	0.12	0.02
	0.63	0.14	0.02
	0.79	0.17	0.02
	0.09	0.37	1.74
	0.07	0.47	1.79
	0.07	0.31	1.68
			0.23
			0.23
			0.24
**Average**	**0.37**	**0.63**	**0.46**
**Standard deviation**	**0.23**	**0.62**	**0.63**

UGT2B15

	Cervix	Uterine	FT
Six separate donors with 3 replicates for each gene. Some fallopian	0.84	0.92	0.78
	0.84	0.95	0.82
	0.79	1.13	0.88
	0.89	0.82	1.00
	0.74	0.82	0.86
	0.70	0.98	1.11
	0.97	0.86	0.73
	0.68	0.62	0.60

tubes donors provided 2 different tubes (left and right)	1.04	0.89	0.96
	0.70	0.79	0.98
	0.82	0.72	0.71
	0.74	0.77	0.73
	1.00	1.11	1.06
	1.07	0.96	0.92
	0.95	0.98	0.99
	0.84	0.54	0.79
	0.88	0.56	1.04
	0.86	0.59	0.70
**Average**	0.85	0.83	0.87
**Standard deviation**	0.12	0.18	0.15

UGT2B17

	Cervix	Uterine	FT
Six separate donors with 3 replicates for each gene. Some fallopian tubes donors provided 2 different tubes (left and right)	0.29	0.98	1.54
	0.28	1.12	1.49
	0.27	1.16	1.45
	0.17	0.63	1.82
	0.17	0.53	1.81
	0.17	0.59	1.89
	0.14	1.57	0.88
	0.16	1.51	0.86
	0.15	1.58	0.80
	1.64	0.90	0.67
	1.68	0.80	0.71
	1.56	0.83	0.66
	1.88	1.32	1.50
	2.07	1.40	1.53
	1.73	1.38	1.32
		1.31	2.57
		0.94	2.81
		0.99	2.00
**Average**	0.82	1.09	1.46
**Standard deviation**	0.80	0.34	0.63
